# Crystal structure of ethyl 4-[(1*H*-pyrazol-1-yl)meth­yl]benzoate

**DOI:** 10.1107/S1600536814025100

**Published:** 2014-11-26

**Authors:** Ju-Xian Wang, Chao Feng

**Affiliations:** a Institute of Medicinal Biotechnology, Chinese Academy of Medical Sciences and Peking Union Medical College, Beijing 100050, People’s Republic of China; b School of Chemistry and Chemical Engineering, Southeast University, Nanjing 210096, People’s Republic of China

**Keywords:** crystal structure, ester, pyrazole derivative

## Abstract

In the title mol­ecule, C_13_H_14_N_2_O_2_, the dihedral angle between the pyrazole and benzene ring mean planes is 76.06 (11)°, and the conformation of the ethyl side chain is *anti* [C—O—C—C = −175.4 (3)°]. In the crystal, the only directional inter­actions are very weak C—H ⋯π inter­actions involving both the pyrazole and benzene rings, leading to the formation of a three-dimensional network.

## Related literature   

For a related structure, see: Dong *et al.* (2011 ▶). For background to the properties of pyrazole derivatives, see: Adnan & Tarek (2004 ▶); Ashraf *et al.* (2003 ▶).
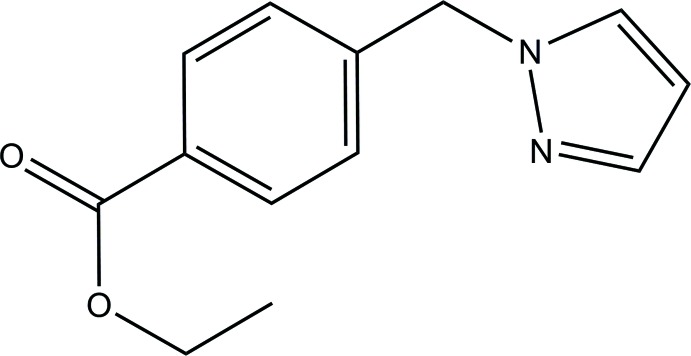


## Experimental   

### Crystal data  

C_13_H_14_N_2_O_2_
*M* *_r_* = 230.26
Triclinic, 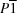

*a* = 8.1338 (12) Å
*b* = 8.1961 (9) Å
*c* = 10.7933 (11) Å
α = 74.013 (9)°
β = 83.308 (10)°
γ = 64.734 (13)°
*V* = 625.54 (13) Å^3^
*Z* = 2
Mo *K*α radiation
μ = 0.08 mm^−1^
*T* = 293 K
0.22 × 0.20 × 0.18 mm

### Data collection  

Agilent SuperNova (Single source at offset, Eos) diffractometer
Absorption correction: multi-scan (*CrysAlis RED*; Agilent, 2012 ▶) *T*_min_ = 0.982, *T*_max_ = 0.985
4295 measured reflections
2197 independent reflections
1639 reflections with *I* > 2σ(*I*)
*R*_int_ = 0.032

### Refinement  

*R*[*F* ^2^ > 2σ(*F* ^2^)] = 0.067
*wR*(*F* ^2^) = 0.201
*S* = 1.15
2197 reflections
155 parameters
H-atom parameters constrained
Δρ_max_ = 0.18 e Å^−3^
Δρ_min_ = −0.23 e Å^−3^

### 

Data collection: *FRAMBO* (Bruker, 2004 ▶); cell refinement: *SAINT* (Bruker, 2004 ▶); data reduction: *SAINT*; program(s) used to solve structure: *SHELXTL* (Sheldrick, 2008 ▶); program(s) used to refine structure: *SHELXTL*; molecular graphics: *SHELXTL*; software used to prepare material for publication: *SHELXTL*.

## Supplementary Material

Crystal structure: contains datablock(s) I, New_Global_Publ_Block. DOI: 10.1107/S1600536814025100/hb7319sup1.cif

Structure factors: contains datablock(s) I. DOI: 10.1107/S1600536814025100/hb7319Isup2.hkl

Supporting information file. DOI: 10.1107/S1600536814025100/hb7319Isup3.cml

**Fig. 1.**
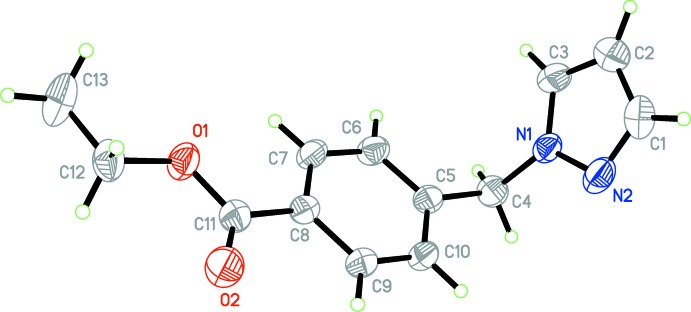
The molecular structure of the title compound with displacement ellipsoids drawn at the 30% probability level.

**Fig. 2.**
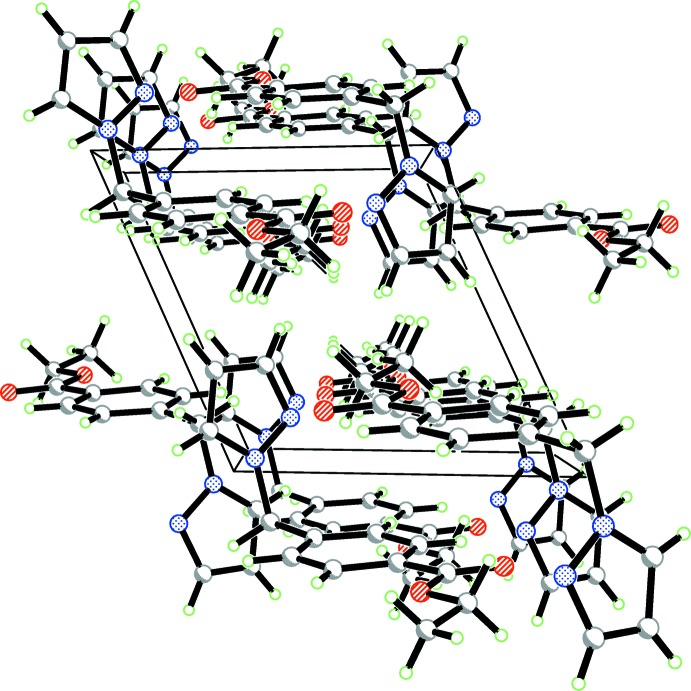
View of the packing diagram of the title compound along the b axis.

. DOI: 10.1107/S1600536814025100/hb7319fig1.tif
The mol­ecular structure of the title compound with displacement ellipsoids drawn at the 30% probability level.

b . DOI: 10.1107/S1600536814025100/hb7319fig2.tif
View of the packing diagram of the title compound along the *b* axis.

CCDC reference: 1034364

Additional supporting information:  crystallographic information; 3D view; checkCIF report

## Figures and Tables

**Table 1:** Hydrogen-bond geometry (, ) *Cg*1 and *Cg*2 are the centroids of the N1/N2/C1C3 and C5C10 rings, respectively.

*D*H*A*	*D*H	H*A*	*D* *A*	*D*H*A*
C2H2*Cg*2^i^	0.93	2.94	3.670(4)	137
C4H4*A* *Cg*1^ii^	0.97	3.00	3.600(3)	122
C12H12*A* *Cg*2^iii^	0.97	2.82	3.689(4)	150

Symmetry codes: (i) 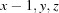; (ii) 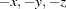; (iii) 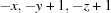.

## supplementary crystallographic information

### S1. Comment

Pyrazole and its derivatives are an important class of *N*-heterocyclic compounds because they exhibit a broad spectrum of pharmacological activities such as antifungal (Adnan & Tarek, 2004), antitumor and antiangiogenic activities (Ashraf *et al.*, 2003). As part of pyrazole derivatives, the crystal structure of 1, 4-bis[(1*H*-pyrazol-1-yl)methyl]benzene has been determined (Dong *et al.*, 2011). As part of this ongoing search for new pyrazole compounds, the title compound was synthesized and its crystal structure is reported herein. In the title compound (Fig. 1), bond lengths and angles fall in normal ranges. The dihedral angle between the pyrazole ring (N1/N2/C1—C3) and the phenyl ring (C5—C10) is 76.06 (11) °. In the crystal, there exists weak C–H···*π* contacts.

### S2. Experimental

In a 250 ml four-necked round-bottom flask equipped with a mechanical stirrer, pyrazole (0.680 g 10 mmol), potassium carbonate (2.073 g 15 mmol) and 1-(4-(bromomethyl)phenyl)-1-hydroxypentan-2-one (2.712 g, 10 mmol) were cautiously dissolved in acetone (100 ml). The solution was heated at 65 °C for 6 h, then the mixture was filtered off was removed by rotatory evaporator at 35 °C and the crude product was obtained 1.815 g (66.1%). Colourless blocks of the title compound were obtained from ethanol by slow evaporation.

### S3. Refinement

H-atoms were placed in calculated positions and refined constrained to ride on their parent atoms, with C—H = 0.93–0.97 Å, *U*_iso_(H) = 1.5*U*_eq_(C) for methyl and 1.2*U*_eq_(C) for the methylene.

### Crystal data

**Table:**

C_13_H_14_N_2_O_2_	*Z* = 2
*M**_r_* = 230.26	*F*(000) = 244
Triclinic, *P*1	*D*_x_ = 1.222 Mg m^−^^3^
Hall symbol: -P 1	Mo *K*α radiation, λ = 0.71073 Å
*a* = 8.1338 (12) Å	Cell parameters from 1485 reflections
*b* = 8.1961 (9) Å	θ = 4.3–28.2°
*c* = 10.7933 (11) Å	µ = 0.08 mm^−^^1^
α = 74.013 (9)°	*T* = 293 K
β = 83.308 (10)°	Block, colourless
γ = 64.734 (13)°	0.22 × 0.20 × 0.18 mm
*V* = 625.54 (13) Å^3^	

### Data collection

**Table:**

Agilent SuperNova (Single source at offset, Eos) diffractometer	2197 independent reflections
Radiation source: fine-focus sealed tube	1639 reflections with *I* > 2σ(*I*)
Graphite monochromator	*R*_int_ = 0.032
phi and ω scans	θ_max_ = 25.0°, θ_min_ = 3.0°
Absorption correction: multi-scan (*CrysAlis RED*; Agilent, 2012)	*h* = −9→9
*T*_min_ = 0.982, *T*_max_ = 0.985	*k* = −9→9
4295 measured reflections	*l* = −12→8

### Refinement

**Table:**

Refinement on *F*^2^	Primary atom site location: structure-invariant direct methods
Least-squares matrix: full	Secondary atom site location: difference Fourier map
*R*[*F*^2^ > 2σ(*F*^2^)] = 0.067	Hydrogen site location: inferred from neighbouring sites
*wR*(*F*^2^) = 0.201	H-atom parameters constrained
*S* = 1.15	*w* = 1/[σ^2^(*F*_o_^2^) + (0.079*P*)^2^ + 0.2197*P*] where *P* = (*F*_o_^2^ + 2*F*_c_^2^)/3
2197 reflections	(Δ/σ)_max_ < 0.001
155 parameters	Δρ_max_ = 0.18 e Å^−^^3^
0 restraints	Δρ_min_ = −0.23 e Å^−^^3^

### Special details

**Table:**

Geometry. All e.s.d.'s (except the e.s.d. in the dihedral angle between two l.s. planes) are estimated using the full covariance matrix. The cell e.s.d.'s are taken into account individually in the estimation of e.s.d.'s in distances, angles and torsion angles; correlations between e.s.d.'s in cell parameters are only used when they are defined by crystal symmetry. An approximate (isotropic) treatment of cell e.s.d.'s is used for estimating e.s.d.'s involving l.s. planes.
Refinement. Refinement of *F*^2^ against ALL reflections. The weighted *R*-factor *wR* and goodness of fit *S* are based on *F*^2^, conventional *R*-factors *R* are based on *F*, with *F* set to zero for negative *F*^2^. The threshold expression of *F*^2^ > σ(*F*^2^) is used only for calculating *R*-factors(gt) *etc*. and is not relevant to the choice of reflections for refinement. *R*-factors based on *F*^2^ are statistically about twice as large as those based on *F*, and *R*- factors based on ALL data will be even larger.

### Fractional atomic coordinates and isotropic or equivalent isotropic displacement parameters (Å^2^)

**Table:**

	*x*	*y*	*z*	*U*_iso_*/*U*_eq_	
C1	−0.3321 (5)	0.2354 (4)	0.0922 (3)	0.0772 (9)	
H1	−0.4412	0.3219	0.0516	0.093*	
C2	−0.3185 (4)	0.0903 (5)	0.1964 (3)	0.0787 (9)	
H2	−0.4119	0.0606	0.2394	0.094*	
C3	−0.1386 (4)	−0.0001 (4)	0.2228 (3)	0.0718 (9)	
H3	−0.0841	−0.1068	0.2885	0.086*	
C4	0.1384 (4)	0.0519 (4)	0.1369 (3)	0.0653 (8)	
H4A	0.1766	0.0861	0.0486	0.078*	
H4B	0.2091	−0.0805	0.1712	0.078*	
C5	0.1753 (3)	0.1603 (4)	0.2163 (2)	0.0543 (7)	
C6	0.2157 (4)	0.0834 (4)	0.3464 (3)	0.0610 (7)	
H6	0.2269	−0.0374	0.3842	0.073*	
C7	0.2396 (4)	0.1856 (4)	0.4205 (2)	0.0584 (7)	
H7	0.2671	0.1326	0.5077	0.070*	
C8	0.2227 (3)	0.3657 (3)	0.3656 (2)	0.0505 (6)	
C9	0.1870 (4)	0.4413 (4)	0.2343 (3)	0.0573 (7)	
H9	0.1784	0.5611	0.1959	0.069*	
C10	0.1643 (4)	0.3379 (4)	0.1609 (3)	0.0588 (7)	
H10	0.1413	0.3888	0.0731	0.071*	
C11	0.2393 (4)	0.4838 (4)	0.4420 (3)	0.0561 (7)	
C12	0.2906 (4)	0.5017 (5)	0.6500 (3)	0.0726 (9)	
H12A	0.1747	0.6050	0.6552	0.087*	
H12B	0.3805	0.5512	0.6164	0.087*	
C13	0.3460 (6)	0.3720 (6)	0.7790 (4)	0.1028 (13)	
H13A	0.2574	0.3219	0.8103	0.154*	
H13B	0.3544	0.4378	0.8374	0.154*	
H13C	0.4622	0.2722	0.7729	0.154*	
N1	−0.0526 (3)	0.0895 (3)	0.13866 (19)	0.0554 (6)	
N2	−0.1716 (4)	0.2383 (3)	0.0558 (2)	0.0741 (8)	
O1	0.2758 (3)	0.3958 (3)	0.56635 (18)	0.0672 (6)	
O2	0.2213 (3)	0.6418 (3)	0.3986 (2)	0.0823 (7)	

### Atomic displacement parameters (Å^2^)

**Table:**

	*U*^11^	*U*^22^	*U*^33^	*U*^12^	*U*^13^	*U*^23^
C1	0.075 (2)	0.072 (2)	0.083 (2)	−0.0205 (17)	−0.0233 (17)	−0.0225 (17)
C2	0.070 (2)	0.088 (2)	0.079 (2)	−0.0365 (18)	0.0043 (16)	−0.0178 (18)
C3	0.078 (2)	0.075 (2)	0.0547 (17)	−0.0352 (17)	−0.0020 (14)	0.0029 (15)
C4	0.0684 (18)	0.083 (2)	0.0557 (17)	−0.0358 (16)	0.0119 (13)	−0.0318 (15)
C5	0.0541 (15)	0.0664 (17)	0.0469 (15)	−0.0276 (13)	0.0101 (11)	−0.0209 (12)
C6	0.0804 (19)	0.0549 (16)	0.0506 (16)	−0.0330 (14)	0.0032 (13)	−0.0109 (12)
C7	0.0788 (19)	0.0601 (16)	0.0391 (14)	−0.0336 (14)	0.0003 (12)	−0.0086 (12)
C8	0.0534 (14)	0.0530 (14)	0.0456 (14)	−0.0235 (12)	0.0016 (11)	−0.0114 (11)
C9	0.0660 (17)	0.0557 (15)	0.0489 (15)	−0.0289 (13)	−0.0030 (12)	−0.0035 (12)
C10	0.0630 (17)	0.0721 (18)	0.0404 (14)	−0.0305 (14)	0.0011 (11)	−0.0090 (13)
C11	0.0593 (16)	0.0558 (16)	0.0552 (16)	−0.0262 (13)	−0.0016 (12)	−0.0124 (13)
C12	0.079 (2)	0.082 (2)	0.072 (2)	−0.0358 (17)	−0.0023 (15)	−0.0388 (17)
C13	0.131 (3)	0.105 (3)	0.070 (2)	−0.031 (2)	−0.027 (2)	−0.039 (2)
N1	0.0692 (14)	0.0607 (13)	0.0401 (12)	−0.0296 (11)	−0.0015 (10)	−0.0136 (10)
N2	0.100 (2)	0.0673 (16)	0.0533 (14)	−0.0374 (14)	−0.0200 (13)	−0.0007 (12)
O1	0.0917 (15)	0.0665 (12)	0.0502 (12)	−0.0353 (11)	−0.0060 (10)	−0.0181 (9)
O2	0.1173 (19)	0.0621 (13)	0.0756 (15)	−0.0452 (13)	−0.0147 (13)	−0.0102 (11)

### Geometric parameters (Å, º)

**Table:**

C1—N2	1.327 (4)	C7—H7	0.9300
C1—C2	1.367 (5)	C8—C9	1.391 (3)
C1—H1	0.9300	C8—C11	1.487 (4)
C2—C3	1.351 (4)	C9—C10	1.385 (4)
C2—H2	0.9300	C9—H9	0.9300
C3—N1	1.333 (4)	C10—H10	0.9300
C3—H3	0.9300	C11—O2	1.200 (3)
C4—N1	1.448 (3)	C11—O1	1.336 (3)
C4—C5	1.524 (4)	C12—O1	1.460 (3)
C4—H4A	0.9700	C12—C13	1.481 (5)
C4—H4B	0.9700	C12—H12A	0.9700
C5—C10	1.381 (4)	C12—H12B	0.9700
C5—C6	1.385 (4)	C13—H13A	0.9600
C6—C7	1.386 (4)	C13—H13B	0.9600
C6—H6	0.9300	C13—H13C	0.9600
C7—C8	1.384 (4)	N1—N2	1.348 (3)
			
N2—C1—C2	112.2 (3)	C10—C9—C8	119.9 (2)
N2—C1—H1	123.9	C10—C9—H9	120.1
C2—C1—H1	123.9	C8—C9—H9	120.1
C3—C2—C1	104.4 (3)	C5—C10—C9	120.9 (2)
C3—C2—H2	127.8	C5—C10—H10	119.5
C1—C2—H2	127.8	C9—C10—H10	119.5
N1—C3—C2	108.4 (3)	O2—C11—O1	122.7 (3)
N1—C3—H3	125.8	O2—C11—C8	124.4 (3)
C2—C3—H3	125.8	O1—C11—C8	112.9 (2)
N1—C4—C5	111.3 (2)	O1—C12—C13	107.1 (3)
N1—C4—H4A	109.4	O1—C12—H12A	110.3
C5—C4—H4A	109.4	C13—C12—H12A	110.3
N1—C4—H4B	109.4	O1—C12—H12B	110.3
C5—C4—H4B	109.4	C13—C12—H12B	110.3
H4A—C4—H4B	108.0	H12A—C12—H12B	108.5
C10—C5—C6	119.0 (2)	C12—C13—H13A	109.5
C10—C5—C4	120.7 (2)	C12—C13—H13B	109.5
C6—C5—C4	120.3 (2)	H13A—C13—H13B	109.5
C5—C6—C7	120.4 (2)	C12—C13—H13C	109.5
C5—C6—H6	119.8	H13A—C13—H13C	109.5
C7—C6—H6	119.8	H13B—C13—H13C	109.5
C8—C7—C6	120.4 (2)	C3—N1—N2	110.7 (3)
C8—C7—H7	119.8	C3—N1—C4	127.7 (2)
C6—C7—H7	119.8	N2—N1—C4	121.4 (2)
C7—C8—C9	119.2 (2)	C1—N2—N1	104.3 (2)
C7—C8—C11	122.5 (2)	C11—O1—C12	117.0 (2)
C9—C8—C11	118.3 (2)		
			
N2—C1—C2—C3	−0.8 (4)	C7—C8—C11—O2	−178.2 (3)
C1—C2—C3—N1	0.6 (4)	C9—C8—C11—O2	1.1 (4)
N1—C4—C5—C10	−88.7 (3)	C7—C8—C11—O1	1.5 (4)
N1—C4—C5—C6	89.5 (3)	C9—C8—C11—O1	−179.2 (2)
C10—C5—C6—C7	1.8 (4)	C2—C3—N1—N2	−0.3 (4)
C4—C5—C6—C7	−176.5 (2)	C2—C3—N1—C4	174.4 (3)
C5—C6—C7—C8	0.2 (4)	C5—C4—N1—C3	−89.3 (4)
C6—C7—C8—C9	−2.0 (4)	C5—C4—N1—N2	84.9 (3)
C6—C7—C8—C11	177.3 (2)	C2—C1—N2—N1	0.6 (4)
C7—C8—C9—C10	1.6 (4)	C3—N1—N2—C1	−0.2 (3)
C11—C8—C9—C10	−177.7 (2)	C4—N1—N2—C1	−175.3 (2)
C6—C5—C10—C9	−2.1 (4)	O2—C11—O1—C12	1.0 (4)
C4—C5—C10—C9	176.1 (2)	C8—C11—O1—C12	−178.7 (2)
C8—C9—C10—C5	0.4 (4)	C13—C12—O1—C11	−175.4 (3)

### Hydrogen-bond geometry (Å, º)

Cg1 and Cg2 are the centroids of the N1/N2/C1–C3 and C5–C10 rings, respectively.

**Table:**

*D*—H···*A*	*D*—H	H···*A*	*D*···*A*	*D*—H···*A*
C2—H2···*Cg*2^i^	0.93	2.94	3.670 (4)	137
C4—H4*A*···*Cg*1^ii^	0.97	3.00	3.600 (3)	122
C12—H12*A*···*Cg*2^iii^	0.97	2.82	3.689 (4)	150

Symmetry codes: (i) *x*−1, *y*, *z*; (ii) −*x*, −*y*, −*z*; (iii) −*x*, −*y*+1, −*z*+1.
