# Supplementary figures and images for: Crystal structure of ethyl 4-[(1H-pyrazol-1-yl)meth­yl]benzoate
Source: Acta Crystallogr Sect E Struct Rep Online. 2014 Nov 26;70(Pt 12):o1287. doi: 10.1107/S1600536814025100 (PMC4257449; doi:10.1107/S1600536814025100)

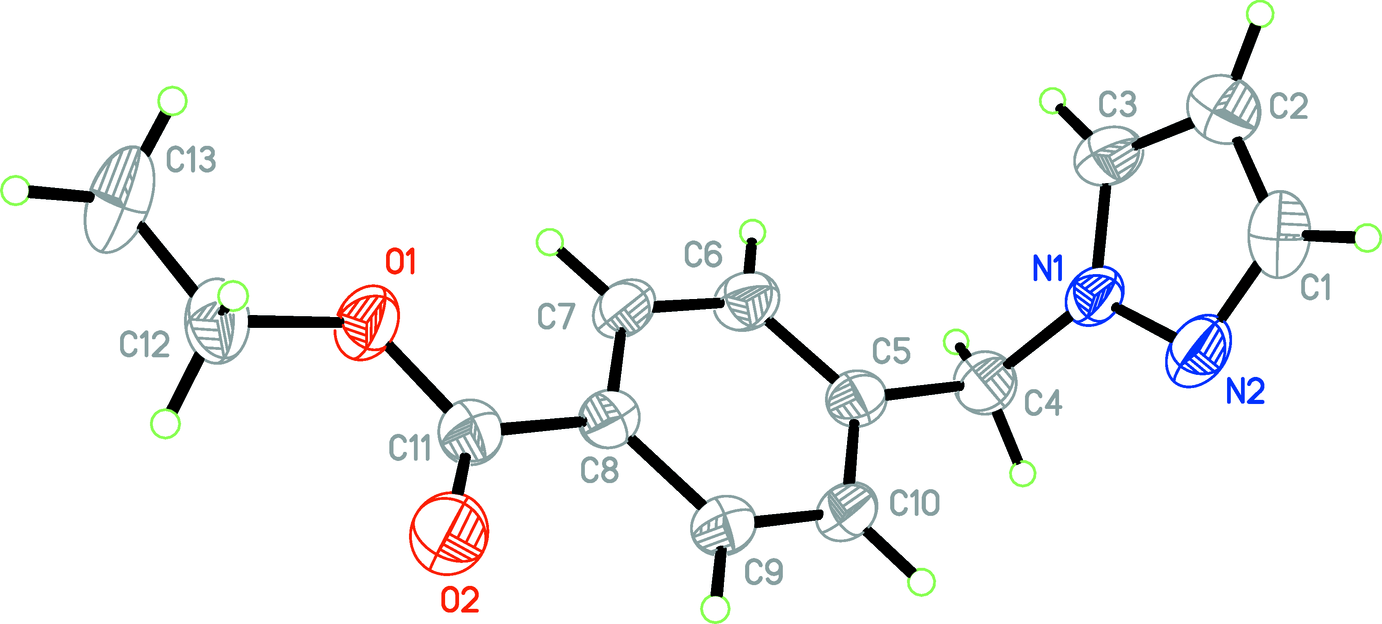

Supplement: Supplementary file 4 [file e-70-o1287-fig1.tif]

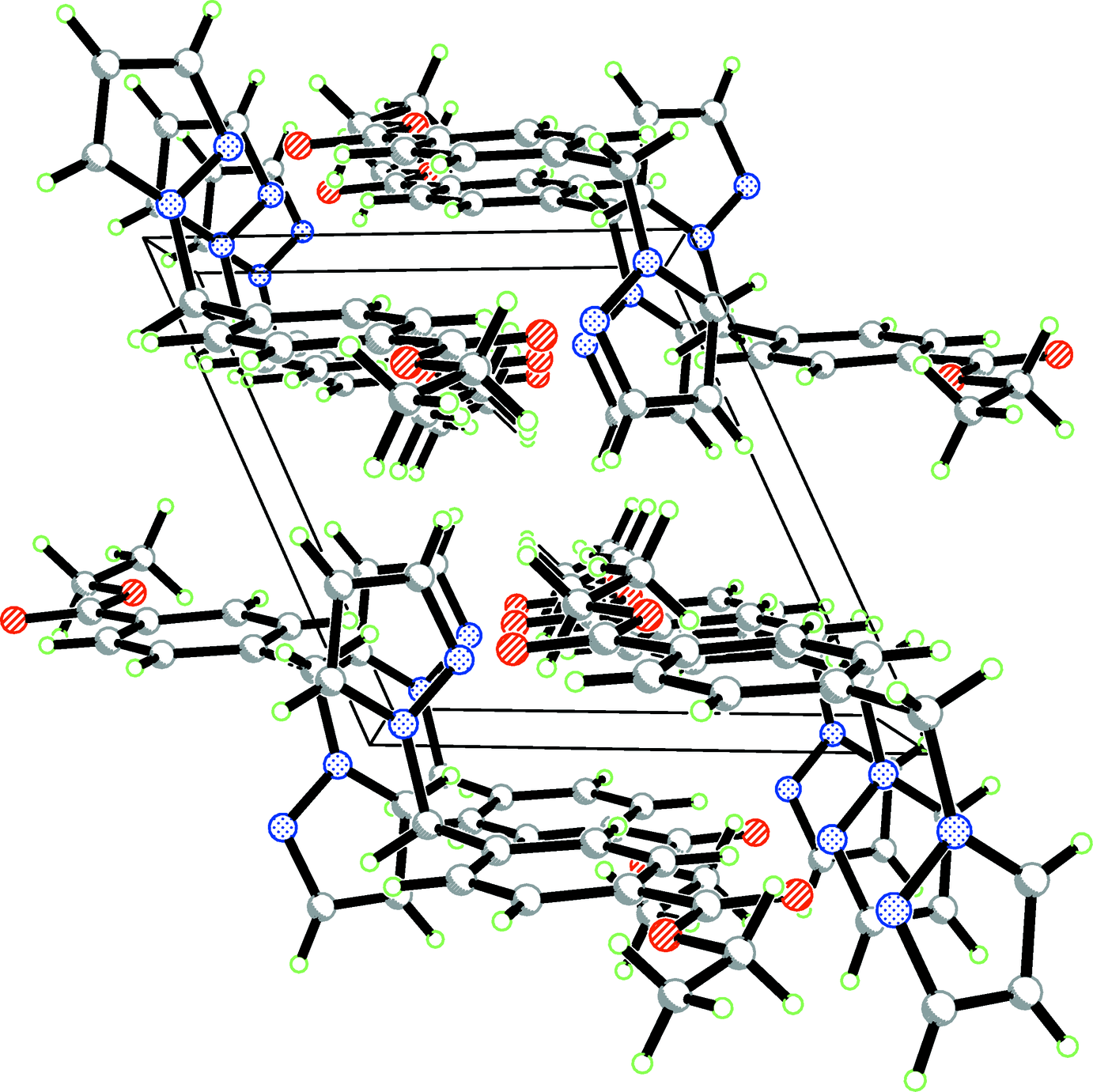

Supplement: Supplementary file 5 [file e-70-o1287-fig2.tif]
